# Impact of Climate‐Smart Crop Intensification on Rural Household Food Security in North Wollo Zone, Ethiopia

**DOI:** 10.1002/pei3.70062

**Published:** 2025-06-06

**Authors:** Getnet Zeleke Tessera, Sisay Demeke Molla

**Affiliations:** ^1^ Department of Geography and Environmental Studies Woldia University Woldia Ethiopia; ^2^ Department of Political Science and International Relations Woldia University Woldia Ethiopia

**Keywords:** climate‐smart, endogenous switching regression model, food security, intensification

## Abstract

Land degradation and climate change are interconnected environmental pressing challenges that significantly contribute to declining agricultural productivity and worsening food insecurity in Ethiopia. To address these challenges, the Ethiopian government introduces climate‐smart agricultural practices, including drought‐tolerant and early‐maturing crop varieties, small‐scale irrigation practices, and efficient fertilizer use. This study examined the impact of climate‐resilient crop intensification strategies on household food security, measured by household food consumption score (HFCS), household dietary diversity score (HDDS), and household food insecurity access scale (HFIAS). The data were collected from 411 smallholder farmers using structured questionnaires, focus group discussions, and key informant interviews. The multistage sampling technique was employed to select study participants. Analysis techniques involved descriptive statistics, the food security index, the ordered probit model, and an endogenous switching regression model. The study reveals the multidimensional nature of household food security: 87.83% of households have better food access (HFCS), 56.45% have moderate dietary quality (HDDS), yet 70.8% experience food insecurity (HFIAS), highlighting persistent access challenges. Adopting all three climate‐smart crop intensification strategies considered in this study, including maturing crop varieties, small‐scale irrigation practices, and efficient fertilizer use, significantly improves household food consumption and dietary diversity while reducing food insecurity. Joint adoption of these strategies increases food variety by 90.5% and decreases food insecurity by 69.9%. Effective extension services, irrigation infrastructure, and viable crop varieties are crucial for enhancing adoption rates and improving food security. The findings of this study emphasized the importance of integrating multiple climate‐smart agricultural practices to enhance food security in Ethiopia. By adopting a combination of drought‐tolerant crops, small‐scale irrigation, and efficient fertilizer use, smallholder farmers can significantly improve their household food consumption and dietary diversity while reducing food insecurity. It is recommended that smallholder farmers adopt a combination of climate‐smart strategies to enhance crop productivity and food security, supported by strengthened extension services that provide implementation guidance.

## 1 Introduction

Agriculture is a crucial economic sector and the primary source of employment in Ethiopia, similar to most sub‐Saharan African countries. Historically, agriculture has been a significant contributor to the Ethiopian economy, often accounting for around 42% of the gross domestic product (GDP) and employing about 80% of the workforce (Bachewe et al. 2018; FAO 2016; Teklewold et al. 2013). However, by 2023, the share of agriculture in the country's GDP had decreased to approximately 32% (African Development Bank 2024; Statista 2024; Tesfaye et al. 2023). Despite this, the sector accounts for approximately 54.1% of total household food consumption (Komarek et al. 2019), with small‐scale rainfed farming dominating 95% of the national annual crop production (Asfaw Eshetu and Mekonen 2024; Eshete et al. 2020; FAO 2016; Fantaye 2016). The sector continues to struggle with low productivity, reliance on rainfed systems, low agricultural inputs, and poor market access (AKLDP 2017; Ejeta and Bai 2025; Teferi et al. 2025; World Bank 2016).

In Ethiopia, land degradation and climate change are significant threats to agricultural production and food security (Admassie and Abebaw 2021; Gebreselassie et al. 2016; Nelson et al. 2009; Tadesse and Hailu 2024; World Bank 2012). Agricultural land expansion at the expense of forestland and shrublands is a critical sustainability issue in Ethiopia driven mainly by population growth and its different economic demands (Berihun et al. 2019; Betru et al. 2019; Gebreselassie et al. 2016; Hailu et al. 2020; Miheretu and Yimer 2018). Moreover, little application of improved production technology in Ethiopia during the past three decades has resulted in declining per capita food production and devastating environmental degradation, leading to chronic dependence on food aid (Borlaug 2007; Diriba 2018; Fikire and Emeru 2022; Hawas and Degaga 2023; Spielman et al. 2011; Waje et al. 2024; Zegeye et al. 2022). Therefore, ensuring food security requires both maintaining environmental sustainability and enhancing agricultural productivity (Ajibade et al. 2023; Haggar et al. 2021; Garnett et al. 2013; Mishenin et al. 2021).

Sustainable intensification has been proposed to achieve food security and reduce agriculture's environmental impacts by narrowing yield gaps on existing agricultural land while improving resource use efficiencies (Ajibade et al. 2023; Coomes et al. 2019; Gondwe et al. 2024; Lal 2015; Rockström et al. 2017; Silva et al. 2021; Snyder and Sulle 2022). In the past three decades, Ethiopia has enormously boosted agricultural productivity via modern inputs and intensification and stimulated overall economic development. It also envisioned an agricultural‐led industrialization (ADLI) strategy and implemented agricultural development plans that mainly focused on transforming its agriculture sector. Improving cereal production and intensification through improved use of fertilizer, improved seeds, and agrochemicals (chemical products used in agriculture, which include pesticides, fertilizers, and insecticides, led by an extensive public extension system) has been taken as a core pillar of a series of agricultural development strategies implemented in subsequent years (Yu et al. 2011). Inorganic fertilizer intensification has been considered a fundamental game changer in Ethiopia's transformation agenda to address the productivity loss caused by soil fertility loss (Berhane et al. 2022). As a result, fertilizer imports have more than doubled over the last two decades (Berhane et al. 2020).

Like fertilizers, improved seed intensification has been at the leading edge of Ethiopia's public funding of agricultural studies and extension services drive to increase cereal production over the last two decades (Spielman et al. 2012a, 2012b). The nationally cultivated area covered by improved seeds increased substantially, rising from 4.7% in 2007/8 to 13% in 2016/17 (Bachewe et al. 2018). In the 2021 growing season, for instance, maize seed utilization had reached 81.4% (Belachew et al. 2022; Kalsa et al. 2024). As of 2022, the utilization of improved seeds for major crops such as maize, wheat, teff, and sorghum stood at 59%, 48%, 13%, and 2%, respectively (Ethiopian Ministry of Agriculture 2022). Adopting drought‐tolerant and early‐maturing crop varieties, using irrigation, and making efficient fertilizer applications in Ethiopia are strongly advocated as part of building climate‐resilient green economy policy frameworks (Schmidt and Thomas 2020). In the past ten years, Ethiopia's national agricultural research institutes released about 50 varieties of wheat and at least 20 varieties each for maize, barley, and tef (ATA 2017). The proportion of farmers using improved seeds purchased (and the area covered by improved seeds) has seen significant improvements, with more than a doubling noted for teff, barley, wheat, and maize over the past decade (Berhane et al. 2020).

The study examines the impact of adopting climate‐smart crop intensification, including drought‐tolerant and early‐maturing crop varieties, chemical fertilizers, and small‐scale irrigation practices, on smallholder rural household food security in northern Ethiopia. While previous research has focused on maize and wheat, this study highlights tef and sorghum, which are vital in the region but less studied (Georgise et al. 2019). This study fills a gap in research, as previous studies have primarily concentrated on other crops. Evidence suggested that improved agricultural technology adoption positively impacts productivity and food security (Asfaw et al. 2012; Hailu et al. 2014, 2021; Mulugeta and Hundie 2012; Shiferaw et al. 2014; Zegeye et al. 2022).

Ethiopia faces significant agricultural challenges, including low productivity and high vulnerability to climate change, which exacerbate land degradation and food insecurity (Admassie and Abebaw 2021; Bekele et al. 2020; Gebreselassie et al. 2016; Mekonnen and Gokcekus 2020). The adoption of climate‐smart agriculture (CSA) practices is critical for improving food security and resilience among smallholder farmers (Belay et al. 2023; Nazifi 2024). Ethiopia has implemented strategies like the Climate‐Resilient and Green Economy Strategy to address climate change impacts, emphasizing the need for CSA practices to enhance agricultural productivity and food security (FDRE 2019).

Focusing on locally demanded climate‐smart agricultural practices (CSA), the study enables the depiction of the specific needs of smallholder farmers, who are crucial for Ethiopia's food security (Amelework et al. 2016). In this context, few studies have examined adoptions of the input packages focused on tef row planting and land and water management technologies (Cafer and Rikoon 2018). While previous research has explored factors influencing the adoption of such practices, it lacks empirical evidence on their effects on household food security. The study focuses on locally demanded practices like drought‐resistant crops, irrigation, and soil fertility management practices, which are crucial for informing policymakers and agricultural experts on which technologies to scale up or improve. Overall, the study contributes to the broader goal of sustainable intensification, which aims to depict the productivity benefits while reducing environmental impacts (Silva et al. 2021; Coomes et al. 2019). Therefore, the current study investigated the impact of climate‐smart crop intensification practices on household food security in north Wello zones, Ethiopia.

## 2 Methods and Materials

### 2.1 Description of the Study Area

**FIGURE 1:**
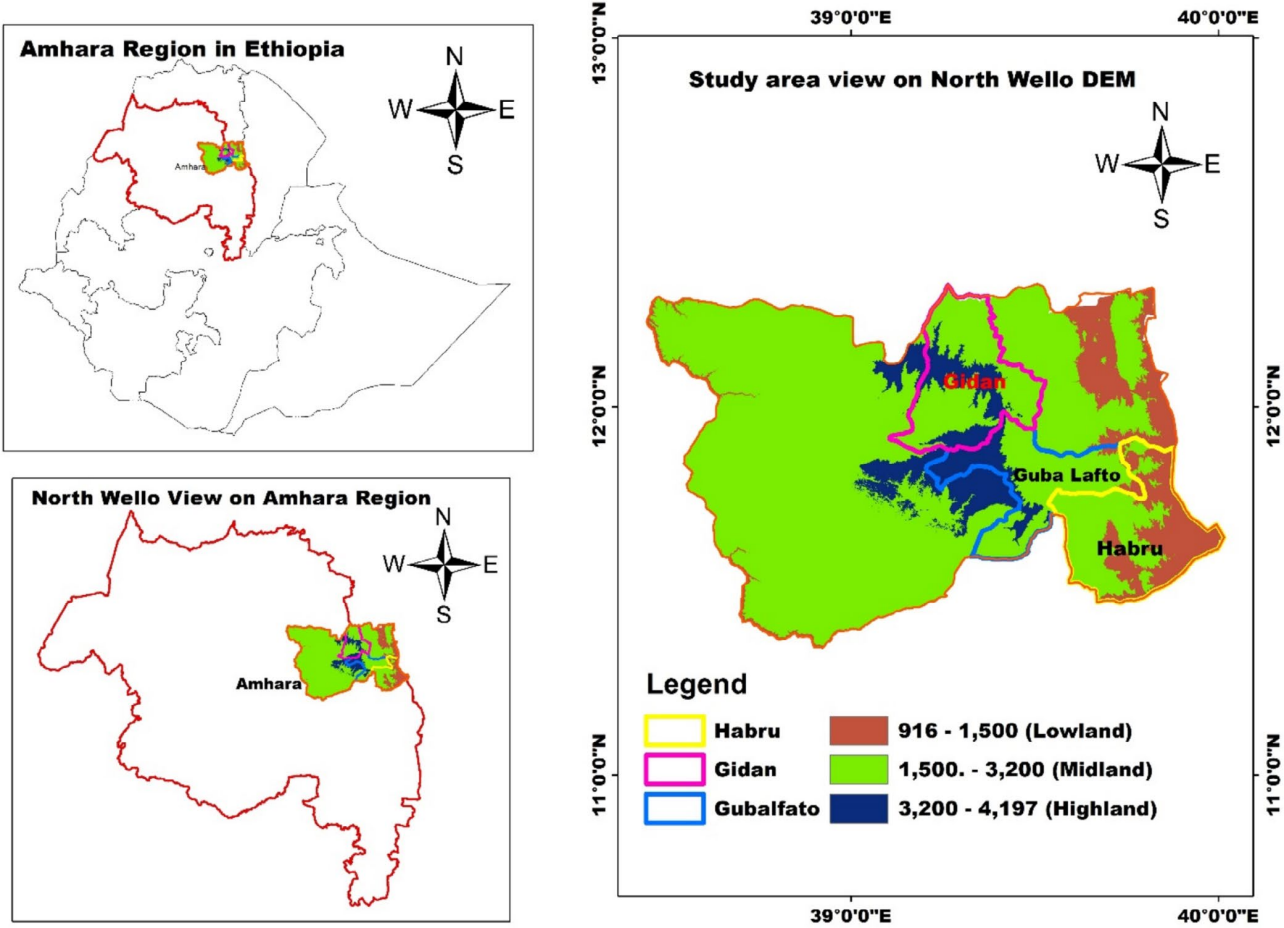
Study area map (accessed from https://www.diva‐gis.org/gdata).

The study was conducted in the North Wollo Administrative Zone, one of the most drought‐prone areas in Ethiopia. The area is located between 11°30′0″ and 12°30′0″ N latitude and 38°30′0″ and 40°0′0″ E longitude (Figure 1). Its elevation ranges from 900 to 4265 m above sea level, encompassing three major agroecological zones: hot (lowland), temperate (middle), and cool (highland) (Eyasu 2016; Gorfu and Ahmed 2012).

**FIGURE 2:**
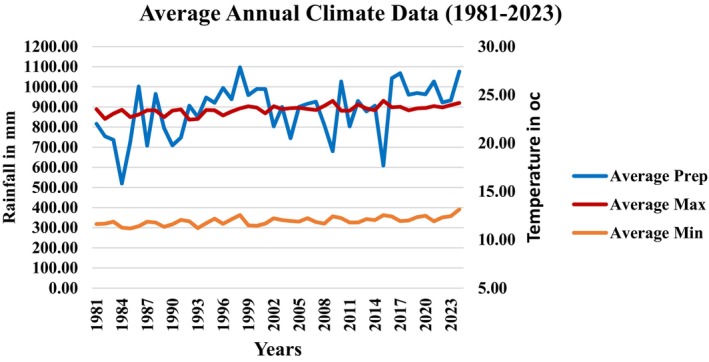
Climate conditions of the study area. *Source:* CHIRPS.

The North Wollo zone is distinguished by a distinct bimodal precipitation pattern, Belg in April–May prior to the primary wet season, Kermit, from July to September (Conway 2000). Based on CHIRPS (Climate Hazards Groups Infrared Precipitation with Station Data) analysis from the period 1981–2013 at 0.05° spatial resolution, precipitation patterns in the study area show marked variability. Annual rainfall fluctuated significantly, with notable peaks during the late 1990s and intermittent dry periods. However, no sustained upward or downward trend emerged, highlighting persistent irregularity in seasonal and annual totals (Figure 2).

In contrast, temperature trends reveal more consistent behaviors. Maximum temperatures remained stable, consistently hovering near 25°C with minor inter‐annual variations. Conversely, minimum temperature demonstrates a gradual upward trend, climbing from approximately 15°C in the earlier years to 17°C–18°C by 2023 (Figure 2). The combination of erratic rainfall and rising baseline temperatures poses dual challenges for agricultural planning and ecosystem management, necessitating adaptations to unpredictable water availability.

**FIGURE 3:**
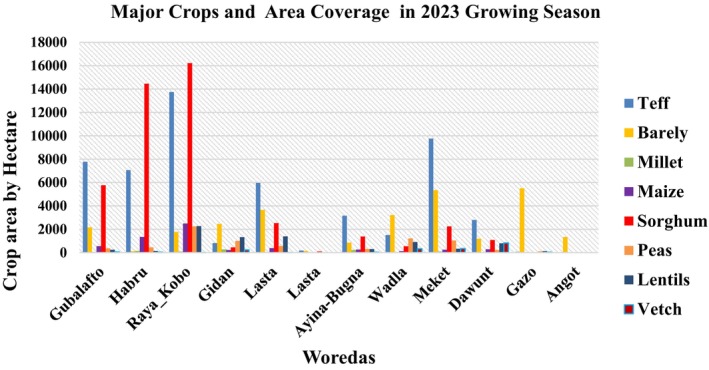
Major crops grown in north Wello zone. *Source:* North Wollo Agriculture Office, 2023.

In the study area, the dominant soils are diverse, including Cambisols, Luvisols, Vertisols, Xerosols, Leptosols, Regosols, and Nitisols. Cereals dominate the agricultural landscape, covering 85.06% of cultivated land, with teff, wheat, sorghum, barley, and maize being the primary crops (Figure 3). These five grains account for about three‐quarters of the total cultivated area, forming the backbone of the local food economy (Taffesse et al. 2012). Beyond cereals, pulses like beans, peas, lentils, and chickpeas also play a significant role, occupying 14% of the cultivated area in 2020–21. The agricultural sector, heavily reliant on grain production, is crucial for both food security and income generation. Additionally, livestock is integral to the farming system, primarily used for plowing and transportation, underscoring the interconnected nature of agriculture and animal husbandry in the region.

### 2.2 Research Methods

#### 2.2.1 Research Approach, Design, and Justification

This study employed a mixed‐methods approach, combining quantitative surveys with qualitative insights to comprehensively analyze farm household dynamics (Creswell 2015). Centered on rural livelihoods, it gathered numerical data from household surveys while capturing nuanced perspectives through interviews and focus group discussions. Using a concurrent embedded design, the research equally prioritized both quantitative data and enriched them with qualitative context, a strategy that strengthened validity through data triangulation. This dual‐layered methodology enabled multi‐level analysis, revealing both statistical patterns and human experiences to address complex agricultural challenges effectively. By integrating Creswell's (2015) design, the approach balanced rigor with adaptability, offering a mixed understanding of farming systems rarely achieved through single‐method studies.

#### 2.2.2 Sampling Design and Procedures

The study relied on primary data sets gathered from rural households via cross‐sectional survey methods. We used a multistage sampling method to select household heads who could participate in the study and to select the *kebeles* from which data would be collected. First, rural administration districts are classified according to their agroecology. Second, three representative districts were purposefully chosen to represent the three dominant agroecological areas to ensure representation across different ecological conditions. Habru, Gubalafto, and Gidan were selected to represent lowland, midland, and highland agro‐ecologies, respectively. This selection was based on recommendations from the agricultural head office in the study zone, which identified these districts as exemplary locations for implementing best agricultural practices. Habru's lower elevation and hotter climate make it an ideal representative of lowland agro‐ecologies. Gubalafto, with its varied topography and bimodal rainfall pattern, is well‐suited as a midland representative. Gidan, situated in the highlands, faces challenges such as crop losses and climate vulnerability, making it a suitable highland representative (Zeleke et al. 2023).

Third, in consultation with agronomic experts in the study area, the *kebeles* that had the dominant characteristics of each sampled district were identified and aggregated based on their dominant agroecological features. This third step is crucial to exclude *kebeles* that do not share the prevailing microclimatic and agricultural characteristics of the sampled districts. Following this, six kebeles, which were two *kebeles* from each sampling district, were selected using a random sampling technique. About 421 randomly selected farm households in the three districts using proportional sampling methods were interviewed from March to June 2023.

#### 2.2.3 Data Sources and Data Collection Methods

The study is based on cross‐sectional household survey data of 421 smallholder farmers conducted from March to June 2023, which coincides with the Belg (small rainy season) and the beginning of the Meher (long rainy season) in North Wollo, Ethiopia. This period is critical for crop planting and growth, making it a suitable time to assess the impact of climate‐smart crop intensification on food security. Collecting data during this period allows for an assessment of how farmers prepare for and manage their crops during the transition from the Belg to the Meher season, which is crucial for understanding their food security strategies. By capturing data during this transition, we can better understand how climate‐smart practices influence food security outcomes across different agricultural zones in North Wollo. The household survey was substantiated by focus group discussions, key informant interviews, and field observations. The household and plot‐level data were collected through structured survey questionnaires addressing household demographics, socioeconomic characteristics, and farmers' use of climate‐smart crop intensification and food security measurement modules. The plot‐level data included the biophysical characteristics of the plot, the type of crop planted in each plot, and the climate‐smart crop intensification applied to each plot. These data were mainly used to determine how climate‐smart crop intensification affects the food security of farm households.

The survey questionnaire used for this study was carefully prepared to ensure it efficiently captured the necessary information related to food security indicators, including the Household Food Consumption Score (HFCS), the Household Dietary Diversity Score (HDDS), and the Household Food Insecurity Access Scale (HFIAS) based on existing literature and guidelines (Coates et al. 2007; Maxwell et al. 2014; Swindale and Bilinsky 2006; Vaitla et al. 2015). Focus group discussions were also carried out to corroborate the responses acquired through the questionnaire. Finally, secondary data, particularly climate data, were also used to supplement primary data collected through questionnaires, critical informant interviews, and focus group discussions.

To ensure the survey questionnaire's reliability and validity, it was pre‐tested among local farmers to identify and address any ambiguities. Based on the feedback, the questionnaire was refined before its finalization. Trained enumerators, familiar with the local language (Amharic) and culture, were trained before the data collection. These enumerators, selected from high school teachers, underwent comprehensive training on the survey tools and ethical considerations, including maintaining neutrality and accurate response recording.

#### 2.2.4 Variables Considered in the Analysis

##### 2.2.4.1 Treatment Variable

In this study, the treatment variables are specific climate‐smart crop intensification practices used by households to enhance food security amidst climate change. These practices include the use of drought‐tolerant and early‐maturing crop varieties, which help farmers adapt to changing rainfall patterns (Wekesa et al. 2018); inorganic fertilizer application to boost crop yields while considering environmental impacts (Zeweld et al. 2015; Wekesa et al. 2018); and small‐scale irrigation techniques to manage water efficiently on small agricultural lands (Tesfaye et al. 2008; Wekesa et al. 2018). The treated group consists of households that have adopted these practices, benefiting from improved crop yields and resilience. Conversely, the untreated group includes households that have not adopted these climate‐smart practices, serving as a control group to compare outcomes.

##### 2.2.4.2 Outcome Variable

The outcome variable used in this analysis is household food security status measured in the household food consumption scale, food dietary diversity scale, and food insecurity access scale.

##### 2.2.4.3 Explanatory Variables

The explanatory variables inputted in the model specification were included based on the theoretical framework and empirical evidence from the literature (Asrat and Simane 2017; Asfaw et al. 2018; Beyene et al. 2017; Fentie and Beyene 2019; Kassie et al. 2015; Teklewold et al. 2017; Tsige et al. 2020). These include demographic factors (education, age, gender, and household size), plot characteristics (plot number, farm distance, slope, soil, and tenure), asset‐related factors (farm size, TLU, and irrigable land), infrastructure, and institutional support variables (Table 1).

**TABLE 1:** Explanatory variables included in the endogenous switching regression model.

Variable	Description	Mean	SD
Gender	Sex of the household head	0.87	—
Age	Age in years of the household head	46.9	9.74
Education	Education status of the household head	0.35	—
Family size	Household size of the respondents	5.56	2.02
Social membership	Household head membership of social organization (Edir, Equb, etc.)	0.88	—
TLU	Livestock herd size in tropical livestock unit	4.01	2.18
Farm size	Total farm size that the farmers owned in hectares	1.10	0.56
Parcel number	Number of plots of cultivated land	2.94	1.18
Gentle slope	The slope of farmland is perceived as moderate in ha	0.37	0.35
Steep slope	The slope of farmland is perceived as very steep in ha	0.12	0.24
Land rent	Sharing of cropland from another farm	0.314	—
Farm distance	Walking distance from home to farmland in minutes	37.7	19.38
Media access	The presence of a radio in the home	0.817	—
Extension	Distance from home to extension services in minutes	47.62	36.91
Market	Distance from home to nearest market in minutes	99.64	125.24
Credit	Credit services available to households	0.70	—
Training	Access to agricultural training for household heads	0.42	—
Climate information	Climate information is obtained from extension services and other media	0.25	—
Age dependency	The proportion of household members under 15 and over 64 compared to those between 15 and 64	2.08	1.52

*Source:* Authors survey, 2023.

#### 2.2.5 Data Analysis Techniques

To analyze the impact of climate‐smart crop intensifications on food security, a robust statistical approach was employed. Descriptive and inferential statistics were utilized to analyze and test the relationships between variables using software tools such as Stata SE14, SPSS version 22, and Microsoft Excel. Key indices such as the Household Food Consumption Score (HFCS), the Household Dietary Diversity Score (HDDS), and the Household Food Insecurity Access Scale (HFIAS) were computed to assess food security among users and non‐users of climate‐smart crop management practices. An ordered probit regression model identified determinants of household food security, while an endogenous switching regression model in Stata using the Movestay command evaluated the effects of the treatment variable (climate‐smart crop intensification) on the outcome variables (HFCS, HDDS, and HFIAS). In addition, qualitative data on the theme under investigation were analyzed thematically simultaneously with the quantitative data and followed by comparison, triangulation, and integration for validation.

#### 2.2.6 Computing Food Security Indices

To capture food security comprehensively requires multiple indicators, as no single measure can encompass all aspects—food availability, access, utilization, and stability. Some indicators, like HDDS and FCS, focus on the quantity and quality of food consumed, while others, such as HFIAS and CSI, assess consumption sufficiency and psychological aspects of food insecurity (Barrett 2010; Coates et al. 2007; Coates 2013; Maxwell et al. 2014; Swindale and Bilinsky 2006). To provide comprehensive perspectives, this study combines HFCS, HDDS, and HFIAS, leveraging their complementary strengths to address both objective and experiential dimensions of food security (Vaitla et al. 2015).

##### 2.2.6.1 Food Consumption Score (FCS)

The food consumption score (FCS) measures dietary diversity and frequency of consumption developed by the World Food Programme (Wiesmann et al. 2009). The food consumption score indicator measures households' food security status by collecting data on typical household diets and incorporating consumption frequency over the past seven days (Vaitla et al. 2012; Wiesmann et al. 2009). HFCS was measured by its weighted score based on food frequency and the nutritional value of the food groups consumed (World Food Programme 2008).

Therefore, the HFCS was calculated by multiplying each food group's frequency by each food group's weight and then summing these scores into one composite score. Finally, according to WFP (2008), the food consumption status of the household was grouped into discrete categories, which can also be expressed as ordinal scores of poor (0–21), borderline (21.5–35), and acceptable (> 35).

##### 2.2.6.2 Household Dietary Diversity Score (HDDS)

The household dietary diversity score (HDDS) measures a variety of food groups over a specific period (FAO 2011). Based on Swindale and Bilinsky's (2006) guidelines, this study used a 24‐h recall of 12 food groups: cereals, tubers, vegetables, fruits, meat, eggs, fish, beans, dairy products, fats and oils, sugar or honey, and condiments. Each consumed group earned one point, resulting in a score from 0 to 12. The scores were categorized into three levels: low (≤ 3), medium (4–6), and high (≥ 7), reflecting varying degrees of dietary diversity (Huluka and Wondimagegnhu 2019). This categorization helps to assess the nutritional quality and diversity of household diets.

##### 2.2.6.3 Household Food Insecurity and Access Scale (HFIAS)

The Household Food Insecurity and Access Scale (HFIAS) was created to assess household behaviors that indicate insufficient food quality and quantity, anxiety, and uncertainty about insecure access or food supply (Coates et al. 2007). According to Coates et al. (2007), respondents were asked nine questions about their food insecurity occurrences and frequency over a four‐week recall period. The frequency‐of‐question occurrences is asked as a follow‐up to each occurrence question to define how frequently the situation occurred. Suppose the respondents answer “yes” to an occurrence question. In that case, they are then asked whether the condition infrequently occurred (once or twice), occasionally (three to ten times), or frequently (more than ten times) in the previous four weeks.

The HFIAS score continuously measures the household's food insecurity (access) over the previous four weeks (30 days). First, the HFIAS score variable is calculated for each household by adding the codes for each frequency‐of‐occurrence question. Following Coats et al.'s (2007) guidelines, all frequency‐of‐occurrence codes were summed, and the frequency of occurrence was coded as 0 for all cases where the answer to the corresponding occurrence question was “no.” The maximum score for a household is 27 (the response to all nine frequency‐of‐occurrence questions was “often,” coded with a response code of 3); the minimum score is 0. The higher the score, the greater the household's food insecurity (access). The lower the score, the less food insecurity a household experience (Coats et al. 2007). Then, the four HFIAS categories were created sequentially to ensure that households are classified according to their most severe response from the Household Food Insecurity Access Scale (HFIAS) generic questions.

Food secure = 1 if (I1 = 0 or I1 = 1) and I2 = 0 and I3 = 0 and I4 = 0 and I5 = 0 and I6 = 0 and I7 = 0 and I8 = 0 and I9 = 0

Mild food insecure = 2 if (I1 = 2 or I1 = 3 or I2 = 1 or I2 = 2 or I2 = 3 or I3 = 1 or I4 = 1 and I5 = 0 and I6 = 0 and I7 = 0 and I8 = 0 and I9 = 0)

Moderate food insecure = 3 if (I3 = 2 or I3 = 3 or I4 = 2 or I4 = 3 or I5 = 1 or I5 = 2 or I6 = 1 or I6 = 2 and I7 = 0 and I8 = 0 and I9a = 0)

Severity food insecure = 4 if (I5 = 3 or I6 = 3 or I7 = 1 or I7 = 2 or I7 = 3 or I8 = 1 or I8 = 3 or I9 = 1 or I9 = 2 or I9 = 3)

The study by Mango et al. (2014) described that HFIAS is the perfect method because it includes all four areas of food security: access, anxiety, inadequate quality, and quantity of food supply.

##### 2.2.6.4 Ordered Probit Model

The ordered probit regression model was applied to analyze the influence of socioeconomic, institutional, and natural capital on food security measured by HFCS, HDDS, and HFIAS indicators. The food security indicators used in the study (HFCS, HDDS, and HFIAS) are categorical and ordinal; therefore, the ordered probit regression model is more suitable for the analysis (Cordero‐Ahiman et al. 2020; Greene 2012; Samim et al. 2021). The ordered probit regression model is specified for HFIAS as follows:

$$\gamma_{i}^{*}=\mathrm{Xi\beta}+\varepsilon_{i}$$

where γ* is household food security with HFIAS categories (food secure, mildly food insecure, moderately food insecure, and severely food insecure); it is a vector of observed non‐randomized explanatory variables determining household food insecurity; and ε is an error term with mean 0 and variance 1. Then, the values for the observed variable *γ*_*i*_ are assumed to be related to the latent variable in the following manner (Samim et al. 2021).

$$\gamma_{i}=\left(\begin{array}{l} 1\;\text{if}\;\gamma_{i}^{*}\leq \mu_{2}\;\text{food secure}\\ 2\;\text{if the}\;\mu_{2}<\gamma_{i}^{*}\mu_{3}\;\text{mildly food insecure}\\ 3\;\text{if the}\;\mu_{3}<\gamma_{i}^{*}\mu_{4}\;\text{moderately food insecure}\\ 4\;\text{if the}\;\gamma_{i}^{*}>\mu_{4}\;\text{severely food insecure} \end{array}\right)$$

The *μ* shows the threshold or cut‐points to be predicted for any category. Thus, as indicated by Samim et al. (2021), the equation for the likelihood with the observed outcomes for the ordered probit model will be

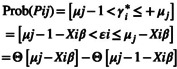

where ϴ is the cumulative distribution function of *εi*; *β* is the regression coefficient for (*Xi*), and *j* is the response categories for HFIAS.

Marginal effects measure the effects of changes in the explanatory variables on cell probabilities. The ordered probit model with *j* alternatives will have *j* sets of marginal effects. Accordingly, the marginal effect of an increase in a regressor *X* on the probabilities of a household falling within *j* response category is given by:

$$\frac{\partial \mathrm{Pij}}{\partial \mathrm{Xi}}=\Theta \;\left[\mathrm{\mu j}-\mathrm{Xi\beta}\right]-\Theta \;\left[\mathrm{\mu j}-1-\mathrm{Xi\beta}\right]\beta$$

where ϴ is the standard normal density function, then following (Samim et al. 2021), the final estimated model is specified as:

HFIJSij=αj+β1X1+β2X2+βnXn+εi

The *αj* parameters, named threshold or breakpoints, are in increasing order (*α*1 < *α*2 < *α*3 < *α*4) (Samim et al. 2021). The order cumulative numbers are *j* = 1, 2, *j* − 1; here, *j* represents the HFIAS ordinal categories (Wooldridge 2010).

##### 2.2.6.5 Endogenous Switching Regression Model (ESRM)

The ESR model was used to estimate the effects of climate‐smart crop intensification on food security. Using the full information maximum likelihood (FIML) estimation technique, we used the ESR model to determine who was more likely to benefit from adopting climate‐smart crop intensification practices.

##### 2.2.6.6 Endogenous Switching Regression (ESR) Model Specification

We used the endogenous switching regression (ESR) model to address these issues. The ESR method specifies separate outcome equations for each regime based on the selection equation. In a two‐stage framework, the ESR model estimates a two‐outcome equation corresponding to users and non‐users. Accordingly, with a random utility conceptual model, it is assumed that farmers are risk‐neutral, and their decisions to use climate‐smart crop intensification practices or non‐use are based on their expected net benefits. Hence, the decisions to use improved seed varieties, inorganic fertilizers, and irrigation both in isolation and in combination in the latent variable framework are specified following Kassie et al. (2014) and Adego et al. (2019) as:

(1) $$\left(\varepsilon_{\mathrm{ij}}\right):U_{i}^{*}=X_{i}\beta_{j}+\varepsilon_{i},$$

where ($X_{i}$) is observed exogenous variables (socioeconomic conditions of farmers and biophysical characteristics of farm plot), β is the vector of the parameters to be estimated, and $\varepsilon_{\mathrm{ij}}$ is unobserved characteristics. The researcher does not observe $U_{i}^{*}$, rather the actual use status can be only observed ($U_{i}$), expressed as

(2) $$U_{i}=\left\{\begin{array}{c} 1,\;\text{if}\;U_{i}^{*}>0\\ 0,\;\text{if}\;U_{i}^{*}<0 \end{array}\right.$$

*U*_*i*_ is a binary dummy variable, which equals 1 for CSA used by the $i^{tk}$ household, and zero, otherwise. Furthermore, due to the household decides to use CSA or not, the yield benefits with CSA and without are specified as an endogenous switching regime model conditional on $\mu_{i},$ like that:

(3) $$\text{users regime}:\;Y_{0i}=\beta_{1}X_{1i}+\mu_{1i},\;\text{if}\;\;\mu_{i}$$

(4) $$\text{non}-\text{users regime}:Y_{0i}=\beta_{0}X_{0i}+\mu_{10},\;\text{if}\;\mu_{i}=0$$

The left‐hand side variables $Y_{1i}\text{and}\;Y_{0i}$ refer to the food security outcome measured by HFCS, HDDS, and HFIAS corresponding to the two regimes' users and non‐users of climate‐smart crop intensification, respectively. On the right‐hand side, $\beta_{1}$ and $\beta_{0}$ are vectors of parameters to be estimated; $X_{1i}$ and $X_{0i}$ are vectors of determinants of crop yield for the $i^{tk}$ household, $\mu_{1i}$, and $\mu_{0i}$ are the error terms. The error terms ε in Equation (1) and the two ($\mu_{1i},\mu_{10}\;)$ in Equations (3) and (4) have a trivariate normal distribution with zero mean and a non‐singular covariance matrix (Adego et al. 2019) expressed as follows:

(5) $$\begin{array}{ccc} \sigma_{1}^{2} & \sigma_{10} & \sigma_{1}\varepsilon \\ \mathrm{Cov}\left(\varepsilon_{i},\mu_{1i},\mu_{0i}\;\right)=\;\sigma_{10} & \sigma_{0}^{2} & \sigma_{0}\varepsilon \\ \sigma_{1}\varepsilon & \sigma_{0}\varepsilon & \sigma_{\varepsilon }^{2} \end{array}$$

where $\sigma_{1}^{2}$= var. ($\mu_{1}$), $\sigma_{0}^{2}$= var. ($\mu_{0}$), $\sigma_{10}$= cov ($\mu_{1i}.\mu_{0}),\sigma_{1}\varepsilon$= cov ($\mu_{1}$. ε), $\sigma_{0}\varepsilon$= cov ($\mu_{0.}\varepsilon$), and $\sigma_{\varepsilon }^{2}$ = 1. The regimes $Y_{1i}$ and $Y_{0i}$ outcomes cannot be simultaneously observed for a farmer; hence, the covariance $\left(\sigma_{10}\right)$ is assumed to be zero (Madalla 1983, cited in Amadu et al. 2020).

Moreover, due to selection bias, the expectations of the error terms in Equations (3) and (4) are different from zero. Applying ordinary least squares (OLS), regression would therefore result in biased estimations of $Y_{1i}$ and $Y_{0i}$ (Lee 1982, cited in Amadu et al. 2020).

Under the above assumption, the truncated error terms $\left[\mu_{1i}/U_{i}=1\right]$ and $\left[\mu_{0i}/U_{i}=0\right]$ have the following expected values:

(6) E[μ1iUi=1]=σ1εϕβXiΦβXi=σ1ελ1i

(7) E[μ0iUi=0]=−σ0εϕβXi1−ΦβXi=σ0ελ0i

where $\lambda_{1i}=\;\frac{\phi \left[\beta X_{i}\right]}{\Phi \left[\beta X_{i}\right]}$ and $\lambda_{0i}=\;\frac{\phi \left[\beta X_{i}\right]}{1-\Phi \left[\beta X_{i}\right]};\phi$ is the standard normal probability distribution, and Φ is the standard normal cumulative distribution $\lambda_{1i}$ and $\lambda_{0i}$ are interpreted as the inverse mill's ratios (Heckman 1979) evaluated at $\beta X_{i}$ and used in Equations (3) and (4) to correct for selectivity bias through the two‐stage estimation techniques. However, this technique produces heteroscedastic residuals that are inefficient in generating consistent standard errors (Lokshin and Sajaia 2004). As a result, the full information maximum likelihood technique produces an efficient ESR output by simultaneously estimating the selection and production equations. Previous studies have widely used this method (Adego et al. 2019; Amadu et al. 2020). It allows for estimating the correlation coefficients between the stochastic terms in the selection and outcome models to determine whether or not endogenous switching is prevalent.

The effect of climate‐smart crop intensification on food security was estimated using an endogenous switching regression model, following the work of Adego et al. (2019), Amadu et al. (2020), Kassie et al. (2014), and Teklewold et al. (2013). The multinomial endogenous switching treatment regression model can be used to compute the counterfactual and average adoption effects. The counterfactual is defined as the food security outcome of users, which could have been obtained if the returns (coefficients) on their characteristics had been the same as the returns (coefficients) on the characteristics of the non‐users of CSA practices, and vice versa (Kassie et al. 2014). From Equations (6) and (6), the following conditional expectations for each outcome variable can be computed:

(8) user withactual:EY1iUi=1=β1X1i+σ1iελ1i

(9) the user withoutactual:EY0iUi=0=β0X0i+σ0iελ0i

whereas if that household had not used CSA (counterfactual case), they would be specified as:

Users had decided not to use (counterfactual):

(10) EY0iUi=1=β0X0i+σ0iελ0i

Non‐user had decided to apply (counterfactual):

(11) EY0iUi=0=β0X0i+σ0iελ0i

Then the average treated impact of yield for those is computed as:

$$\mathrm{ATT}=X_{1i}\left(\beta_{1}-\beta_{0}\right)+\lambda_{1i}\left(\sigma_{1i}\varepsilon -\sigma_{0i}\varepsilon \right),$$

where ATT—represents the average treatment for the treated (with CSA).

The validity of the ESR model needs exclusion restrictions on instrumental variables that can be correlated with the use of crop intensification technologies (positively or negatively) but not with the food security outcome (Adego et al. 2019; Amadu et al. 2020). Thus, the extension services, agricultural technology training, and the farmers' climate information are the instrumental variables that would correlate with the decision to use crop intensification technologies but not directly the food security outcomes.

## 3 Results and Discussion

### 3.1 Descriptive Characteristics of Sampled Respondents

The authors first presented the result of the socio‐demographic characteristics of the farmers in Table 1 before reporting and discussing the impact of climate‐smart crop intensification practices on household food security. Table 1 above shows that the study sample consists primarily of male‐headed households (87%), with an average age of 47 years (SD = 9.74). A significant portion of household heads have no formal education, with only 35% being literate (able to read and write). The average household size is approximately 6 members (SD = 2.02), and the age dependency ratio is 2.08 (SD = 1.52). In terms of socioeconomic factors, the average annual income is 41,067.17 ETB (SD = 19,673.47), and households hold an average of 4.01 tropical livestock units (TLU) (SD = 2.18). The average farm size is 1.10 ha (SD = 0.56), with farmers having cultivated land on approximately 3 plots of land on average (SD = 1.18). Around 31.4% of farmers rent land from other neighborhood farmers. The average walking distance to farmland is 37.7 min (SD = 19.38). Most of the sampled household heads have access to media, with 81.7% of households owning a radio. Credit services are available to 70% of households, while access to agricultural training is less common, with only 42% of household heads having received training. Only 25% of households receive climate information. Regarding institutional factors, 88% of household heads are members of social organizations. The average distance to extension services is 47.62 min (SD = 36.91), and the average distance to the nearest market is 99.64 min (SD = 125.24).

### 3.2 Households' Food Security Status Under Different Agro‐Ecological Zones

The mean values of three food security indicators, such as food consumption score, household dietary diversity score, and food insecurity access scale, were compared across different agroecological zones to evaluate variations in food security status among groups, as shown below.

### 3.3 Household Food Consumption Score

The result indicated that most of the sampled households in the midland (99.4%), lowland (79.5%), and highland (79.2%) had an acceptable food consumption score. While no households in the midland had a poor or inadequate food consumption score, 4.7% in the highland and 3.4% in the lowland faced inadequate food consumption. The findings show that most of the sampled households (87.8%) have an acceptable food consumption score (> 35 points), while 9.7% fall into the borderline category (21.5–35 points), and 2.4% have poor food consumption (< 21 points). Table 2 summarizes these categories.

**TABLE 2:** Household food consumptions score by different agroecology.

Food security	Agroecology			Total	
	Midland (%)	Lowland (%)	Highland (%)	Frequency	%
Poor (0–21)	0.0	3.4	4.7	10	2.4
Borderline (21.5–35)	0.6	17.1	16.1	40	9.7
Acceptable (> 35)	99.4	79.5	79.2	361	87.8
Total	100	100	100	411	100

*Source:* Authors' Field survey, March to June 2023.

#### 3.3.1 Household Dietary Diversity Score

Table 3 shows that 21.2% of the households have high diet diversity (≥ 7 food groups/day), while most (56.4%) fall into the medium category (4–6 groups). However, 22.4% have low diversity (≤ 3 groups), indicating limited dietary variety for a significant portion of households.

**TABLE 3:** Household dietary diversity score by different agroecology.

Food security	Agroecology			Total	
	Midland (%)	Lowland (%)	Highland (%)	Frequency	%
Low (0–3)	13.8	23.9	31.5	92	22.4
Medium (4–6)	53.4	65.9	54.4	232	56.4
High (7–12)	32.8	10.2	14.1	87	21.2
Total	100	100	100	411	100

*Source:* Authors' Field survey, March to June 2023.

#### 3.3.2 Household Food Insecurity Access Scale (HFIAS)

Table 4 reveals that 58.2% of households experienced some level of food insecurity, categorized as mildly (27.3%), moderately (24.3%), or severely food insecure (6.3%). The remaining 41.8% were food secure. Food insecurity was most prevalent in the lowland zone, where only 12.5% of households were food secure, compared to 52.3% in the midland and 47% in the highland. Many households struggled with food access due to limited production, financial constraints, and resource shortages.

**TABLE 4:** Prevalence of household food insecurity.

HFIAS categories	Agroecology			Total	
	Midland (%)	Lowland (%)	Highland (%)	Frequency	%
Food secure	52.3	12.5	47.0	172	41.8
Mildly food insecure	27.0	36.4	22.1	112	27.3
Moderately food insecure	16.7	43.2	22.1	100	24.3
Severely food insecure	4.0	8.0	8.7	27	6.6
Total	100	100	100	411	100

*Source:* Authors' Field survey, March to June 2023.

### 3.4 Determinants of Household Food Security

#### 3.4.1 Household Food Consumption Score

The results of food security conditions measured by the HFCS model are presented in Table 5. The ordered logit model fits the data well (*p* = 0.000); log‐likelihood ratio (*X*2 (88.5) = −132.9; pseudo *R*2 = 0.2498). Since the coefficients of the ordered logit model do not represent the magnitude of the effects of the explanatory variables, it is only discussed in terms of the significant marginal effects. Only the variables that are significant in the marginal effects have been discussed.

**TABLE 5:** Results of ordered probit model for household food consumption score (HFCS).

Variables	HFCS category	Marginal effects		
		Poor	Borderline	Acceptable
	Coeff	Coeff	Coeff	Coeff
Gender	−0.051	0.001	0.003	−0.004
Age	−0.015	0.000	0.001	−0.001
Education	0.844*	−0.019	−0.047*	0.066*
Family size	−0.034	0.001	0.002	−0.003
Social networks	−1.287*	0.029	0.072*	−0.101*
PSNP benefits	−1.035**	0.023*	0.058**	−0.081**
TLU	−0.069	0.002	0.004	−0.005
Farm size	0.703	−0.016	−0.039	0.055
Plot fragmentation	−0.021	0.000	0.001	−0.002
Access to irrigable land	14.739	−0.333	−0.823	1.156
Land rent	0.135	−0.003	−0.008	0.011
Farm distance	−0.008	0.000	0.000	−0.001
Media access	1.011**	−0.023*	−0.056**	0.079**
Market	−0.002*	0.001*	0.001*	−0.001**
Credit	−0.028	0.001	0.002	−0.002
Experience sharing	1.483**	−0.033*	−0.083**	0.116**
Climate information	−0.314	0.007	0.018	−0.025
Age dependency ratio	0.131	−0.003	−0.007	0.010
Off‐farm income	−0.000	0.000	0.000	−0.000
Meher rainfall	0.035*	−0.001*	−0.002*	0.003*
Belg rainfall mn	0.008*	−0.000	−0.001*	0.001*
Constant cut1	4.781			
Constant cut2	6.815			

*Note:* Observations 411.

*Source:* Authors' Field Survey March to June 2023.

* statistically signficant at a level *p* < 0.1.

** statistically sigificant at a level *p* < 0.05.

*** statisticaly significance at a level *p* < 0.01.

In the HFCS model, more educated household heads were 4.7% less likely to fall into the borderline food consumption category and 6.6% more likely to be in the acceptable category compared to their less educated or illiterate counterparts. Although not statistically significant, they were also 1.9% less likely to have poor food consumption. This aligns with Nkomoki et al. (2019), who identified education as a key factor influencing food security in Zambia. Educated household heads are more aware of climate‐smart crop intensification practices and better informed through media, improving household food availability. Additionally, they have better access to get better information on the strategies for producing more on small plots in climate‐smart techniques.

Households with media access were 2.3% less likely to fall into the poor FCS category and 5.6% less likely to be in the borderline category. Conversely, they were 7.9% more likely to be in the good FCS category compared to those without media access. This may be due to their exposure to information on modern agricultural technology and farming systems, which enhance food production for household consumption.

Another notable finding revealed that households receiving Productive Safety Net Program (PSNP) benefits were more likely to fall into poor or borderline FCS categories and less likely to be in the acceptable category. While PSNP provides cash and food transfers, permanent direct support, and risk management components, it may also foster dependence among beneficiaries.

Households farther from market areas were 0.1% more likely to fall into the poor and borderline FCS categories but also had a 0.1% higher chance of being in the acceptable category. Limited market access increases transaction costs for input and output marketing, discouraging the adoption of CSA practices due to higher travel and transportation costs. As a result, distance to markets negatively affects access to modern farm inputs like improved seed and chemical fertilizer (Tekelewold et al. 2012).

Household membership in local social systems is another factor influencing food security. The econometric model estimates that household membership (e.g., Edir, Equb) increases the likelihood of being in the borderline food security category by 7.2%, while households were 2.9 times as likely to fall into the poor food security category and 10% less likely to be in the acceptable food security category (Table 6). Additionally, the marginal effects of farmer‐to‐farmer experience sharing indicate that as participation increases, the probability of falling into the poor or borderline FCS category decreases by 3.3% and 8.3%, respectively, while the likelihood of being in the acceptable category rises by 10.2%. Our findings highlight that adequate Meher rainfall reduced the probability of poor or borderline FCS by 1% and increased the likelihood of an acceptable FCS by 3%.

**TABLE 6:** Results of ordered probit model for household food insecurity access score (HFIAS).

Variables	HFIAS_category	Marginal effects			
		Food secure	Mildly food insecure	Moderately food insecure	Severely food insecure
	Coeff	Coeff	Coeff	Coeff	Coeff
Gender	−0.221	0.019	0.013	−0.0039	−0.028
Age	0.03**	0.002	0.002	−0.000	−0.003
Education	−0.35	0.002**	0.002**	0.001	−0.004**
Family size	0.107	0.029	0.021	−0.006	−0.044
Social networks	−0.28	−0.009	−0.006	0.002	0.013
PNSP benefits	−0.66**	0.023	0.017	−0.005	−0.035
TLU	−0.02	0.054**	0.039**	−0.011	−0.082**
Farm size	−0.370	0.002	0.001	−0.000	−0.003
Plot fragmentation	−0.33***	0.030	0.022	−0.006	−0.046
Own to irrigable land	−1.32***	0.027***	0.019***	−0.005*	−0.041***
Land rent	−0.655***	0.107***	0.078***	−0.022*	−0.162***
Farm distance	−0.022***	−0.041	−0.030	0.008	0.063
Media access	−0.455	0.002***	0.001***	−0.001*	−0.003***
Market distance	0.004**	0.000	0.000	−0.000	−0.000
Credit	0.207	−0.000**	−0.000**	0.000	0.001**
Experience sharing	−0.516*	0.019	0.014	−0.004	−0.029
climate information	0.819***	0.042*	0.030*	−0.009	−0.064*
Age dependency ratio	0.084	−0.07***	−0.05***	0.014	0.101***
Off‐farm income	−0.000	−0.007	−0.005	0.001	0.010
Meher rainfall	0.005	0.071**	0.052**	0.015	−0.109**
Belg rainfall	0.006**	−0.000	−0.000	0.000	0.001
Constant cut1	−2.035	−0.000**	−0.000**	0.000	0.001**
Constant cut2	−0.538				
Constant cut3	2.648				

*Source:* Authors' Field survey, March to June 2023.

* *statistically signficant at a level p* < 0.1.

** *statistically siginficant at a level p* < 0.05.

*** *statistically siginficant at a level p* < 0.01.

#### 3.4.2 Household Food Insecurity Access Scale (HFIAS)

The ordered model for HFIAS fits the data well (*p* = 0.000); log‐likelihood ratio (*x* ^2^(22) = 196.47; pseudo (*R* ^2^ = 0.193)), confirming the ranking of food insecurity categories. Table 6 presents the results, highlighting the key factors influencing rural household food insecurity: education, TLU (tropical livestock units), irrigable land, land rent, media access, credit, climate information, age dependency ratio, and *Meher* season rainfall patterns.

Households with educated household heads were 0.2% more likely to fall into the food secure and mildly food insecure categories and less likely to fall into the severely food insecure. FGD participants noted that literacy helps farmers understand better farming techniques, market trends, and climate risks. A male farmer who is 48 years old reported that “*our children who finish school advise us on modern farming. They tell us what fertilizers to use and warn us about fake seeds in the market*.” Education fosters technological adoption, income diversification, and crisis adaptation, ensuring food security (Kolog et al. 2023; Abdullah et al. 2019; Tefera and Tefera 2014).

Greater livestock numbers reduced the likelihood of moderate or severe food insecurity while increasing food security or having mild food insecurity (Table 6). Livestock serves as a key livelihood asset, providing cash income, nutrition, and resilience during food shortages (Ayele et al. 2020; Samim et al. 2021). A household with irrigable land is less likely to fall into the moderately and severely food insecure, reinforcing irrigation's role in stabilizing food supply (Ayele et al. 2020). Renting additional land also improved food security by enabling crop diversification. Households unable to obtain needed credit were more likely to be moderately and severely food insecure. Credit constraints limit investment in improved seed and inorganic fertilizer, reducing agricultural productivity (Tekelewold et al. 2012).

Moreover, a unit increase in household head access to extension services, such as timely climate‐related information, increases the likelihood of farming households being in the food secure and mild food insecurity categories while decreasing the likelihood of severe food insecurity. This result aligns with our prior expectations, as extension agents should enhance awareness of new agricultural technologies and climate information to promote farmers' food security and well‐being.

A higher dependency ratio negatively impacts food security. Each unit increase in the dependency ratio decreased the probability of being food secure by 6.6% while increasing severe food insecurity by 10% (Table 6). This finding is consistent with earlier research conducted in Afghanistan by Samim et al. (2021). A higher dependency ratio reduces per capita income and consumption, affecting the well‐being of household members. The amount of rainfall was another significant factor influencing food insecurity in farm households. The results indicate that a one‐unit increase in *Meher* rainfall tends to enhance food security by 7.1%. In comparison, such households were 5.2% more likely to be in the mild food insecurity category, 1.5% more likely to be in the moderately food insecure category, and 10.9% less likely to be in the severe food insecurity category. Another important variable affecting food insecurity categories is access to media‐based agricultural information. The results show that households with access to media (such as radio and newspapers) had a 0.2% and 0.3% increased chance of being in the food secure and mildly food insecure categories, respectively. In contrast, these households were 0.1% and 0.3% less likely to be in the moderately and severely food insecure categories, respectively. Media facilitates access to agricultural knowledge and market connections, enhancing productivity.

#### 3.4.3 Determinants of Household Dietary Diversity

The ordered logit model results for the household dietary diversity score (Table 7) indicate a significant model fit (*p* < 0.000). Key factors influencing dietary diversity include family size, farmland size, media access, irrigable land, and seasonal rainfall. A one‐unit increase in family size decreases the likelihood of achieving a high dietary diversity score by 1.8% while increasing the probability of having low dietary diversity by 2.5%. Larger household size, especially those with lower incomes, often face resource constraints, prioritizing necessities such as education and health over dietary diversity (Huluka and Wondimagegnhu 2019). An additional hectare of farmland size increased the likelihood of a high HDDS by 7.9% and decreased the probability of low HDDS by 10.9%. Larger landholdings enable crop diversification, enhancing food variety compared to small farmland size focused primarily on staples (Cordero‐Ahiman et al. 2020; Powell et al. 201; Singh et al. 2019). Cultivating multiple crops in a single plot helps to ensure food security and diversity (Fremmpong et al. 2023). The FGD discussion also revealed that households with more farmland reported growing diverse crops, including fruits, vegetables, and legumes. One of the elderly farmers, 62 years old, stated that “*If we have more land, we can grow different foods. But when land is divided among sons, each gets only a small plot, so they grow just sorghum or teff*.”

**TABLE 7:** Results of ordered probit model for household dietary diversity score (HDDS).

Variables	HDDS		Marginal effects		
	Estimates		Low	Medium	High
	Coeff	SE	Coeff	Coeff	Coeff
Gender	0.667	0.333	−0.093	0.005	0.088
Age	−0.000	0.012	0.000	−0.000	−0.000
Education	−0.128	0.237	0.018	−0.001	−0.017
Family size	−0.112**	0.078	0.25***	−0.0069**	−0.018**
Social networks	−0.069	0.357	0.010	−0.001	−0.009
PSNP benefits	0.513	0.341	−0.072	0.004	0.068
TLU	0.102	0.063	−0.014	0.001	0.014
Farm size	0.372***	0.287	−0.109***	0.029**	0.079***
Plot fragmentation	0.011	0.124	−0.001	0.000	0.001
Land rent	−0.168	0.25	0.023	−0.001	−0.022
Access to irrigable land	2.539***	0.435	−0.355***	0.018***	0.336***
Farm distance	0.002	0.006	−0.000	0.000	0.000
Media access	1.098***	0.321	−0.153***	0.008***	0.145***
Market	−0.001	0.001	0.000	−0.000	−0.000
Credit	0.066	0.251	−0.009	0.000	0.009
Age dependency ratio	−0.012	0.09	0.002	−0.000	−0.002
Off‐farm income	0.001**	0.001	−0.001**	0.001	0.001**
Meher rainfall	−0.014	0.008	0.002*	−0.000	−0.002
Belg rainfall	0.005**	0.002	−0.001***	0.000***	0.001***
Constant cut1	−2.725	2.573			
Constant cut2	−0.080	2.567			

*Note:* Observations 411.

*Source:* Authors' Field survey, March to June 2023.

* *p* < 0.1.

** *p* < 0.05.

*** *p* < 0.01.

In addition, households with media access are 14.5% more likely to achieve high dietary diversity and 15.3% less likely to fall into the low HDDS category. Media provides critical information on agricultural technology, market access, and climate risks, improving household resilience and food security (Harris‐Fry et al. 2015).

Owning irrigable land significantly increases the likelihood of higher diversity, as irrigation supports the production of nutrient‐rich crops such as fruits and vegetables. Key FGD results show that households with irrigable land produced vegetables and fruits year‐round, increasing dietary diversity. A male farmer, who is 50 years old, reported that “*With irrigation, we don't just eat teff and sorghum. We can grow and sell tomatoes, onions, and bananas, improving both income and diet*.” The findings of the study are supported by research conducted in South Africa, Ethiopia, and Tanzania by Taruving et al. (2013), Passarelli et al. (2018), and Mekonnen et al. (2012), who found that irrigation is positively associated with a high dietary diversity score.

According to the estimation result, a 1% increase in *Belg* rainfall improves dietary diversity, making households more likely to have a high HDDS. The FGD participants reported that good Belg rains (short rainy season) allowed the production of early‐maturing crops and vegetables. However, a 1% increase in *Meher* rainfall correlates with lower dietary diversity. This may be due to variations in seasonal crop availability and reliance on staple production.

### 3.5 The Effects of Climate Smart Crop Intensifications on Food Security

In this section, we present the results from the Endogenous Regression Switching Regression (ESR) model estimates. First, we estimate the factors that influence the decision to adopt climate‐smart intensification of crop production technologies. The ESR model estimates two separate but related outcome equations, one for adopters and one for non‐adopters, along with a combined selection equation for the adoption of climate‐smart crop intensification technologies. Second, the treatment effects of adoption on food security indicators of HFCS, HDDS, and HFIAS. However, since our primary objective in this article is to assess the impact of climate‐smart intensification of crop production technologies, we only discuss selected endogenous switching regression estimates here to conserve space.

Table 8 presents the impact of climate‐smart crop intensification on rural household food security status measured by key food security indicators: HFCS, HDDS, and HFIAS. Adopters of improved technologies consistently outperform non‐adopters across all food security indicators. For instance, households using combined agricultural technologies (improved seed, fertilizers, and irrigation) show the highest HFCS (64.5) and HDDS (7.1), along with the lowest HFIAS (3.4), indicating superior food security. The Average Treatment Effect on the Treated (ATT) reveals significant benefits: joint adoption of all three technologies improves HFCS by 17.9%, HDDS by 1.5 points, and reduces HFIAS by 7.9 points, all statistically significant at the 1% level. Conversely, non‐adopters face stark disadvantages, as shown by the Average Treatment Effect on the Untreated (ATU). For example, if non‐adopters adopted irrigation, their HFCS would rise by 18.45 points (from 39.6 to 58.05), highlighting the potential gains from technology uptake.

**TABLE 8:** Treatment effects of CSA practices measured by food security indicators.

Technology set	Adopters (actual)	If they would not adopt	ATT	Non‐adopters	If they would adopt	ATU
Household Food Consumption Score (HFCS)						
Improved seed varieties	50.9	49.61	1.3*	39.1	45.9	−6.9***
Inorganic fertilizers	46.5	38.2	8.3***	30.1	34.4	−4.3***
Irrigation	63.7	52.44	11.32***	39.6	58.05	−18.45***
Improved seed with chemical fertilizers	52.6	42.5	10.1***	39.6	51.3	−11.7***
Use of improved seed, fertilizer, and irrigation jointly	64.5	46.6	17.9***	42.5	64.7	−22.2***
Household Dietary Diversity Score (HDDS)						
Improved seed varieties	6.1	5.5	0.6***	4.5	7.3	−2.8***
Inorganic fertilizers	5.33	4.64	0.7***	4.7	3.6	1.1***
Irrigation	8.0	4.2	3.8***	6.0	6.2	−0.2***
Improved seed with inorganic fertilizers	7.5	5.5	2***	4.7	5.9	−1.2***
Use of improved seed, fertilizer, and irrigation jointly	7.1	5.6	1.5***	4.6	5.9	−1.3***
Household Food Insecurity Access Scale (HFIAS)						
Improved seed varieties	6.78	12.84	−6.06***	11.65	9.49	2.16***
Inorganic fertilizers	7.0	11.87	−4.9***	15.25	11.83	3.42***
Irrigation	3.6	3.8	−0.2***	11.68	7.8	3.88***
Improved seed with inorganic fertilizers	6.5	11.4	−4.9 ***	11.5	10.8	0.7***
Use of improved seed, fertilizer, and irrigation jointly	3.4	11.3	−7.9***	10.7	5.7	5.0***

*Note:* ATT: Average Treatment Effect on the Treated. This measures the effect of the technology on those who actually adopted it. ATU: Average Treatment Effect on the Untreated. This measures the effect if non‐adopters were to adopt the technology.

*Source:* Authors' field survey, March to June 2023.

* Statistically significant at a level < 10%.

*** Statistically significant at a level < 1%.

Additionally, it was found that, on average, adopters of improved seed varieties have 2.6% (1.3%) higher HFCS than their counterparts, and the difference is statistically significant. Additionally, on average, adopters of inorganic fertilizers (DAP and urea) experience a 21.7% (8.3%) increase in HFCS. In contrast, their counterparts showed a 12.5 (−4.3) decrease, and this difference is also statistically significant. Furthermore, adopters of irrigation practices achieve the highest HFCS (63.7) and HDDS (8.0), likely due to enhanced crop yields and year‐round production. However, non‐adopters relying on rainfed systems suffer from lower HFCS (39.6) and higher HFIAS (11.68), underscoring irrigation's role in stabilizing food access. On average, adopters of small‐scale irrigation practices have a 21.6% (11.32) increase in HFCS, whereas non‐adopters recorded a 31.8% (−18.45) fall in HFCS, and the difference is statistically significant. This result is aligned with the previous studies by Zeweld et al. (2015) and Adeniyi and Dinbabo (2020), which found that small‐scale irrigation has a robust and positive effect on most of the livelihood indices in Ethiopia. They also suggest that expanding irrigation schemes is a viable strategy for water‐stressed and drought‐prone areas in Ethiopia.

Farmers who adopt both improved seed and inorganic fertilizers experience a combined 23.76% (10.1) increase in HFCS. In contrast, the non‐adopters record a 22.8% decline in HFCS, and this difference is statistically significant at a level < 1%. Studies by Abay et al. (2018), Berhane et al. (2022), and Ogada and Nyangena (2019) in Ethiopia and Kenya have found similar results, indicating that intensification measures—primarily fertilizer and improved seed varieties—have a positive and significant relationship with the most food security indicators. Furthermore, the results show that the highest impact on HFCS (38.1%) is achieved through the joint adoption of all three climate‐smart crop intensification technologies, which is more significant than the impacts of each technology used in isolation. Previous empirical evidence from Abay et al. (2018), Hailu et al. (2021), and Mengistie and Kidane (2016) in Ethiopia suggests that while using fertilizers and improved seed varieties individually offers some benefits, combining them in both rainfed and irrigated farms can increase yields because they are highly complementary to each other.

The findings also indicate that farm households adopting irrigation practices have significantly increased their households' food diversity by 90.5%. Additionally, results reveal that a combination of improved technology adoption positively and substantially affects the Household Dietary Diversity Score (HDDS). Farm households that adopted a combination of all three improved technologies (irrigation, improved seed, and inorganic fertilizer) significantly increased their household food diversity by 26.8% (1.5). In contrast, the lowest dietary diversity score was obtained (10.9%) when households adopted only improved seed varieties. The study shows that, generally, irrigation practices yield a higher dietary diversity score than any other practices, whether adopted individually or jointly. Access to small‐scale irrigation enabled the sample households to cultivate various crops more than once a year, ensuring improved and steady output, income, and consumption, which in turn enhanced their food security status (Tesfaye et al. 2008).

In addition to this, on average, adopters of all three combined technologies have shown a 69.9% (−7.9) decline in the Household Food Insecurity Access Scale (HFIAS), while their counterfactual showed an 87.7% (5.0) increase, and the difference is statistically significant. Furthermore, adopters of improved seed varieties have reported a 47.2% (−6.06) decrease in HFIAS. In contrast, their counterparts indicated a 22.8% (2.16) increase in HFIAS. Adopters of inorganic fertilizer and adopters of irrigation practices recorded a 41.3% (−4.9) and 42.9% (−6.02) decrease in HFIAS, respectively. Moreover, adopters of improved seeds combined with inorganic fertilizer had a 43.8% (−4.9) decline in HFIAS. However, their counterparts, on average, experienced a 6.5% (0.7) increase in HFIAS, and this result is statistically significant at a level < 1%.

Generally, the highest HFCS and HDDS are observed among adopters of climate‐smart crop intensification, but their average HFIAS is lower than that of non‐adopters. Conversely, non‐adopters typically have low HFCS and HDDS but high HFIAS. Additionally, if adopter households had not adopted climate‐smart crop intensification, they would likely have had low HFCS and HDDS but high HFIAS. On the other hand, if non‐adopters were to adopt climate‐smart crop intensification packages, they would likely experience high HFCS, HDDS, and low HFIAS. The results of the study are consistent with the findings of Hailu et al. (2021), which demonstrated that adopting multiple improved technologies (e.g., heat‐tolerant crops, zero‐tillage, integrated soil management) significantly increased dietary diversity and energy intake, with maximal benefits from combined use. Overall, the greatest benefits are realized when improved crop seeds, inorganic fertilizers, and small‐scale irrigation are practiced in combinations rather than in isolation.

## 4 Conclusions and Recommendations

Land degradation and climate change are two severe environmental issues that mutually reinforce each other. Both significantly impact Ethiopia's low agricultural output and high food insecurity risks. To address these challenges, the Ethiopian government is actively promoting climate‐smart crop intensification measures, such as climate‐resistant high‐yield crop seeds, efficient chemical fertilizer application, and small‐scale irrigation practices. This study examines the effects of these climate‐smart crop intensification measures on rural household food security in the North Wello administrative zone. The study primarily relies on a cross‐sectional survey of farm households, using indicators such as food consumption score, dietary diversity score, and food insecurity access scale to assess the food security status of households. The study highlights the multifaceted nature of food security among rural households, influenced by a combination of socioeconomic, agricultural, and environmental factors.

The study demonstrates a complex interplay of factors influencing food security among rural households. The results, measured by both HFCS and HFIAS models, underscore the importance of multifaceted interventions. Access to resources, information, and productive assets is a key determinant of food security. Key factors include education, as more educated household heads tend to have better food consumption scores and are more adept at adopting new technologies. Access to media and extension services also significantly improves food consumption by providing valuable information on modern agricultural practices. Owning productive assets, such as irrigable land and livestock, reduces food insecurity and enhances dietary diversity. Social capital, particularly through farmer‐to‐farmer experience sharing, positively impacts food security. However, participation in certain social systems can lead to excessive food expenditures. Additionally, safety nets like the Productive Safety Net Program may inadvertently foster dependency. Lastly, proximity to markets is important, as distance can increase transaction costs and limit access to essential inputs and outputs, exacerbating food insecurity.

Results indicate that many farm households adopt climate‐smart crop intensification measures such as climate‐resilient, high‐yielding crop seed varieties, chemical fertilizers, and small‐scale irrigation. The results showed that climate‐smart crop intensification reduces households' food insecurity access scale while significantly increasing household food security, as measured by food consumption and dietary diversity scores. Furthermore, the study found that small‐scale irrigation practices are highly beneficial to achieving household food security, particularly by increasing crop production and enabling access to a more diverse diet. The study demonstrates that the joint adoption of climate‐smart crop intensification measures leads to higher HFCS and HDDS, while reducing HFIAS. Irrigation, improved seeds, and inorganic fertilizers are key drivers of these improvements. Non‐adopters face stark disadvantages, highlighting the need for strategies to promote technology uptake. The outcomes also revealed that climate‐resilient crop intensification measures undoubtedly contribute to achieving Sustainable Development Goals 1, 2, 3, 13, and 15, which focus on ending poverty and hunger, enhancing well‐being, adapting to climate change, and preventing terrestrial land degradation, respectively. Based on the study's findings, the following recommendations are proposed to promote climate‐smart agriculture and enhance food security, along with suggestions for further research.

The combined use of climate‐smart technologies such as small‐scale irrigation, chemical fertilizers, and climate‐resilient crop varieties through subsidies, training programs, and access to credit to maximize food security benefits is strongly encouraged. It is also suggested to prioritize investments in small‐scale irrigation infrastructure, especially in water‐stressed and drought‐prone areas, to significantly impact household food security. It is crucial to design targeted interventions, particularly for non‐adopters, to address barriers like lack of information, access to inputs, and financial constraints. Enhancing agricultural extension services to equip farmers with knowledge and skills is crucial to effectively implementing climate‐smart practices. Future research that should expand upon the current findings by exploring several key areas is very crucial. One important future research direction is to conduct longitudinal studies that assess the long‐term impacts of climate‐smart crop intensification practices on food security. This would provide important insights into how these technologies perform over time and help to identify sustainable solutions. Additionally, incorporating a qualitative research approach would allow for a deeper understanding of the social, cultural, and institutional factors that influence decisions to adopt climate‐smart technologies.

## Conflicts of Interest

The authors declare no conflicts of interest.

## Data Availability

The data that supports the findings of this study are available in the supporting information of this article.
